# Cytomegalovirus Infection of the Rat Developing Brain *In Utero* Prominently Targets Immune Cells and Promotes Early Microglial Activation

**DOI:** 10.1371/journal.pone.0160176

**Published:** 2016-07-29

**Authors:** Robin Cloarec, Sylvian Bauer, Hervé Luche, Emmanuelle Buhler, Emilie Pallesi-Pocachard, Manal Salmi, Sandra Courtens, Annick Massacrier, Pierre Grenot, Natacha Teissier, Françoise Watrin, Fabienne Schaller, Homa Adle-Biassette, Pierre Gressens, Marie Malissen, Thomas Stamminger, Daniel N. Streblow, Nadine Bruneau, Pierre Szepetowski

**Affiliations:** 1 INSERM U901, Marseille, France; 2 Mediterranean Institute of Neurobiology (INMED), Marseille, France; 3 UMR_S901, Aix-Marseille University, Marseille, France; 4 CIPHE (Centre d'Immunophénomique), PHENOMIN, UM2 Aix-Marseille University, Marseille, France; 5 INSERM US012, Marseille, France; 6 CNRS UMS3367, Marseille, France; 7 PPGI platform, INMED, Marseille, France; 8 PBMC platform, INMED, Marseille, France; 9 INSERM, U1141, Paris, France; 10 Paris Diderot University, Sorbonne Paris Cité, Paris, France; 11 PremUP, Paris, France; 12 Institute for Clinical and Molecular Virology, University of Erlangen-Nuremberg, Erlangen, Germany; 13 Vaccine & Gene Therapy Institute, Oregon Health and Science University, Portland, Oregon, United States of America; University of St Andrews, UNITED KINGDOM

## Abstract

**Background:**

Congenital cytomegalovirus infections are a leading cause of neurodevelopmental disorders in human and represent a major health care and socio-economical burden. In contrast with this medical importance, the pathophysiological events remain poorly known. Murine models of brain cytomegalovirus infection, mostly neonatal, have brought recent insights into the possible pathogenesis, with convergent evidence for the alteration and possible involvement of brain immune cells.

**Objectives and Methods:**

In order to confirm and expand those findings, particularly concerning the early developmental stages following infection of the fetal brain, we have created a model of *in utero* cytomegalovirus infection in the developing rat brain. Rat cytomegalovirus was injected intraventricularly at embryonic day 15 (E15) and the brains analyzed at various stages until the first postnatal day, using a combination of gene expression analysis, immunohistochemistry and multicolor flow cytometry experiments.

**Results:**

Rat cytomegalovirus infection was increasingly seen in various brain areas including the choroid plexi and the ventricular and subventricular areas and was prominently detected in CD45^low/int^, CD11b^+^ microglial cells, in CD45^high^, CD11b^+^ cells of the myeloid lineage including macrophages, and in CD45^+^, CD11b^–^ lymphocytes and non-B non-T cells. In parallel, rat cytomegalovirus infection of the developing rat brain rapidly triggered a cascade of pathophysiological events comprising: chemokines upregulation, including CCL2-4, 7 and 12; infiltration by peripheral cells including B-cells and monocytes at E17 and P1, and T-cells at P1; and microglia activation at E17 and P1.

**Conclusion:**

In line with previous findings in neonatal murine models and in human specimen, our study further suggests that neuroimmune alterations might play critical roles in the early stages following cytomegalovirus infection of the brain *in utero*. Further studies are now needed to determine which role, whether favorable or detrimental, those putative double-edge swords events actually play.

## Introduction

Congenital cytomegalovirus (CMV) infections are the leading cause of human neurodevelopmental disorders, with an incidence of about 1% of all live births [1]. Upon maternal infection, CMV can cross the placental barrier and reach the fetus. Overall 5–10% of infected neonates show severe neurological and other defects at birth (*e*.*g*. growth retardation, microcephaly) and another 10% will develop severe neurological diseases (*e*.*g*. hearing loss, cerebral palsy, epileptic seizures, intellectual disability). Also, in the remaining 80–85% the long-term consequences are unknown; the possible influence on various brain disorders such as autism, schizophrenia or epilepsy has been postulated but remains elusive. The severity and the somehow unpredictable outcome of such congenital infections, the difficulties in generating a CMV vaccine [2] and generally the current lack of an effective preventive strategy, the limited efficacy of antiviral neonatal treatment on the severe deficits, are in contrast with the relative paucity in the number of studies actually aiming at deciphering the mechanisms of such brain infections, particularly *in utero*—hence the long-lasting lack of a clear understanding of the underlying pathophysiological processes [3]. Having better knowledge on the pathophysiological mechanisms of congenital CMV infection yet represents a health care and socio-economical priority.

CMVs are large DNA viruses of the *Herpesviridae* family; they show strict species specificity. Several animal models of embryonic or neonatal CMV infection of the brain have been reported [4]. Although CMVs of various species exhibit similarities in genome content as well as in cell and tissue tropisms and in pathological consequences, the exploration of congenital CMV infection in animal models has yet proven to be problematic. Indeed, differences in placental layer organization preclude materno-fetal transmission of CMV infection in rodents. Intraperitoneal, transplacental, or intracerebroventricular routes of CMV inoculation have been used to solve this issue, each having its own advantages and limitations. Notwithstanding the inherent variability of CMV infections, different periods of injection, and various developmental time points, cell types, and possible consequences were studied—hence the difficulties in getting a clear and convergent picture of the pathophysiological mechanisms. Despite those difficulties and discrepancies, recent findings have suggested that inflammatory processes may play an important role in the pathophysiology of brain CMV infection [5]. Notably the existence of brain immune reactions to murine CMV (MCMV) infection has been reported in neonatal mouse models. Such reactions might include the infiltration by mononuclear cells such as T cells and monocytes, and the production of various chemokines and pro-inflammatory cytokines by glial and microglial cells [6]. It has been proposed that the early recruitment of natural killer (NK) cells, of neutrophils and of monocytes initiates clearance of MCMV [7]. A role for a subset of regulatory B-cells that infiltrated CMV-infected brains of young adult mice, in modifying T-cells and microglia responses, has been demonstrated [8]. It was also recently shown that following *in utero* intraplacental MCMV infection, brain macrophages that had infiltrated the fetal brain showed abnormal activation and were the major infected cells [9]. Whereas murine models of brain CMV infection have brought recent insights into the possible pathogenesis of brain CMV infection, alternative models in other species that would take into account *in utero* events are needed to confirm and expand those findings, particularly concerning the early developmental stages following CMV infection of the fetal brain. The generation and the study of more complementary animal models of different types, differing in the CMV strains and animal species, in the developmental period of viral inoculation, and in the experimental design, should also better reflect the huge variability in prenatal events, in brain structural and functional anomalies, and in clinical outcome, associated with the corresponding human congenital CMV infections.

To get more insights into the pathophysiological events, particularly concerning the early developmental stages following infection of the fetal brain and the possible existence of neuroimmune alterations, we have created a model of rat CMV (RCMV) infection *in utero* by intracerebroventricular (icv) injections into the rat brain at embryonic day 15 (E15) and analyzed this model at different developmental stages, from E16 to P1, using a combination of gene expression analysis, immunohistochemistry and multicolor flow cytometry experiments.

## Materials and Methods

### Ethical statement

All animal experimentations were performed in accordance with the French legislation and in compliance with the European Communities Council Directives (2010/63/UE). Depending on the age of the animals, euthanasia were performed after anesthesia with 4% isoflurane with overdose of pentobarbital (120mg/kg) or with decapitation. All surgical procedures were done under anesthesia and all efforts were made to reduce suffering. *In utero* injections were performed in embryos from timed pregnant rats (embryonic Day 15, E15) that were anaesthetized with ketamine (100 mg/kg) / xylazine (10 mg/kg). This study was approved under the French department of agriculture and the local veterinary authorities by the Animal Experimentation Ethics Committee (*Comité d'Ethique en Expérimentation Animale)* n°14 under licence n°01010.02.

### RCMV strain

The rat cytomegalovirus (RCMV) recombinant Maastricht strain (RCMV-∆145-147-gfp) with a green fluorescent protein (GFP) expression cassette was reported previously [10]. Briefly, supernatants of infected rat fibroblasts (RFL-6; ATCC CCL-192) were first spun at 3000 rpm for 20 min to remove cell debris, and then pelleted for 3 h at 14000 rpm (Beckman SW28). The virus pellet was resuspended in Minimal Essential Medium (MEM) culture media. Then, a second centrifugation was performed at 20000 rpm (Beckman SW41) through a sucrose cushion (15% sucrose, 50 mM Tris, 12 mM KCl, 5 mM EDTA) followed by careful resuspension of the virus pellet in VSP buffer (50 mM Tris, 12 mM KCl, 5 mM EDTA). Virus stocks were stored in aliquots at -80°C. Titration of virus stocks was performed by standard plaque assays.

### Intracerebroventricular (icv) RCMV infection

Wistar rats (Janvier Labs, France) were raised and mated at the INMED Post Genomic Platform (PPGI) animal facility and kept at room temperature under conditions of optimal health hygiene. *In utero* injections were performed as previously described [11] in embryos from timed pregnant rats (embryonic Day 15, E15) that were anaesthetized with ketamine (100 mg/kg) / xylazine (10 mg/kg). In each embryo, 1 μl of minimal essential medium (MEM) with Fast Green (2 mg/ml; Sigma) and 5% fetal calf serum, either containing 3.5 10^3^ plaque forming unit (pfu) of RCMV, or not (control), was injected icv *via* pulled glass capillaries and a microinjector (PV 820 Pneumatic PicoPump, World Precision Instruments).

### Immunohistochemistry experiments

Immunohistochemistry experiments were carried out as described previously [11] on coronal brain slices (50–100 μm, vibratome, Microm; 14 μm, cryostat, Leica) with the appropriate primary (Table A in S1 File) and secondary (Alexa Fluor_568 or 647-conjugated goat anti-rabbit, anti-mouse, anti-rat, anti-guinea pig IgGs or donkey anti-goat IgGs; Life Technologies) antibodies. Hoescht 33258 (1:2000, Sigma) was used for nuclei staining.

### Microscopy and cell counting

Images were acquired by using a Stereo Microscope Olympus SZX16 equipped with digital camera DP73, or a Zeiss Axio Imager Z2 microscope with structured illumination (ApoTome) equipped with Zeiss AxioCam MRm camera and processed using Axiovision software, or with a confocal laser scanning microscope Leica TCS SP5X equipped with a light laser, a 405 nm diode for ultra-violet excitation, and 2 HyD detectors. Gross histology and scoring of infected brain areas were performed with binocular stereo microscope. When a given brain region of interest (ROI) displayed GFP staining indicative of active RCMV infection, it was scored as a positive event and used to compute the infection index, meant to indicate the frequency of infection of a given brain area, independently of the number of GFP-labeled cells in this area. For cell counting analyses, at least three adjacent brain sections were analyzed throughout the entire z-dimension for each sample using confocal microscopy. Images were acquired (1024 x 1024 pixels) using the 40x oil-immersion objective (NA 1.30) and were composed of three channels corresponding to GFP (488 nm), Iba1 (568 nm) and ED1 (633 nm). Cells were counted manually in each brain with Image J Software [12] and the associated Cell Counter plugin at E17 and E20, and with Cell Profiler cell image analysis software (Broad Institute Cambridge, MA, USA) at P1. For each channel of stack images, mean image intensity was measured using “MeasureImageIntensity” module and cells were identified with “IdentifyPrimaryObjects” module, in which Min and Max typical diameter of objects was set between 20 and 80 pixel units, respectively, and threshold was corrected by the mean intensity measured previously. Then, using mask images generated with the aforementioned module, colocalization between channels was evaluated with “RelateObjects” and “MaskObjects” in order to determine the number of overlapping objects. Finally, data were exported to a spreadsheet containing the number of quantified cells for each staining and of colocalizations. This pipeline was validated on sample images before applying it to the whole set of experimental pictures. Activation index of microglia in a given ROI was defined as the ratio of the number of Iba1^+^ Ed1^+^ cells to the total number of Iba1^+^ cells. Morphology index of microglia in a given ROI was calculated as the ratio of the number of amoeboid activated, Iba1^+^ cells versus total number of Iba1^+^ cells.

### Quantitative reverse transcription polymerase chain reaction (qRT-PCR)

Total RNAs were extracted from whole embryonic brains (RCMV-infected and control conditions, respectively) taken at various developmental stages (E16, E17, P1) (n = 8 brains per stage in each condition) using TRIZOL reagent according to manufacturer's instructions (Life Technology). cDNA was synthesized from 1 μg of total RNA using Quantitect Reverse Transcription Kit and according to manufacturer protocol (Qiagen). RT-PCRs were then carried out using SYBR-Green chemistry (Roche Diagnostics) and Roche amplification technology (Light Cycler 480). PCR primers (Table B in S1 File) were designed for 11 rat genes encoding classical cytokines (Il-6, Il-1β, Il-10), chemokines (Ccl2, Ccl3, Ccl4, Ccl7, Ccl12), chemokine receptors (Ccr2, Ccr5), and growth factor Tnf-α, for two RCMV genes encoding viral chemokines r129 and r131, and for control gene *Rpl19* (ribosomal protein L19) for relative quantification. All primer pairs were optimized to ensure specific amplification of the PCR product and the absence of any primer dimer. Quantitative PCR standard curves were set up for all. Values of fold change represent averages from duplicate measurements for each sample.

### Flow cytometry

For dissociation into single cell suspension, entire control or CMV-infected brain tissues obtained from embryos at E17, and from anesthetized P1 pups perfused with 2–3 mL of phosphate-buffered saline (PBS), were mechanically homogenized in ice-cold Hank's buffer. Brain homogenates were centrifuged through a continuous 30% Percoll gradient (7750 g, 40 min.) and the bottom phase containing leukocytes was filtered using cell strainers (70 μm). Cells collected in ice-cold Hank's buffer were centrifuged (600g, 5 min.) and suspended in FACS buffer (D-PBS 0.01 M pH 7.4, EDTA 1 mM, BSA 1%). 1 to 3 x10^6^ cells were then incubated with Zombie NIR Fixable Viability kit (1:200; Biolegend) for 20 min at room temperature to discriminate between live and dead cells. Fc receptor were blocked using with mouse anti-rat CD32 antibody (FcγII receptor clone D34-485) for 10 min. at 4°C to prevent against nonspecific binding. Cells were then stained with antibodies against combinations of cell surface markers (Table A in S1 File) in PBS 1% FCS 2 mM EDTA for 45 min. at 4°C, washed twice in FACS buffer, and finally incubated in FACS buffer containg 2% (v/v) PFA. An average of 1.3 x10^5^ living singlet cells were analyzed per embryo equivalent on a BD LSRFortessa (BD Bioscience) cell cytometer and raw data were analyzed using FACSDiva V8.0 software (BD Biosciences).

### Statistics

Data were expressed as mean ± SEM unless otherwise stated. Kruskall-Wallis test followed by Dunn's post-hoc test was used in cell counting analyzes to compare the absolute numbers of infected cells between E17, E20 and P1. Mann-Whitney test (two-tailed) was used in cell counting, in qRT-PCR and in flow cytometry analyzes to compare between RCMV-infected and control brains, with the appropriate adjustment for multiple comparisons (Bonferroni) whenever appropriate.

## Results

### Time course of RCMV infection in the rat fetal brain

**Fig 1:**
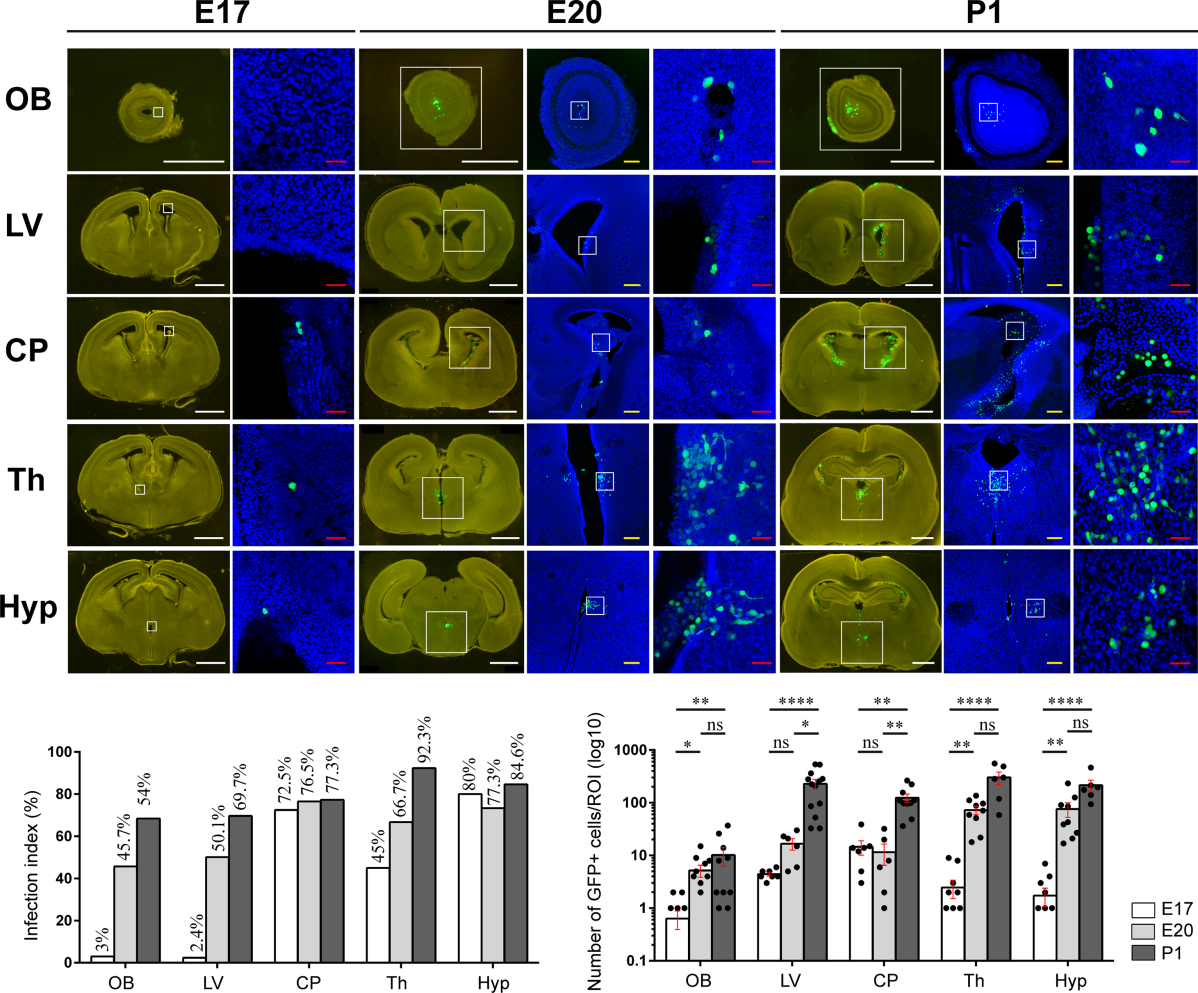
Time course of rat CMV (RCMV) dissemination in the developing brain. Recombinant RCMV allowing expression of GFP (green) in the infected cells, was injected intraventricularly at E15. Brains were analyzed at various developmental time points using fluorescent binocular microscopy (left panel of each corresponding stage) and confocal microscopy (right panels of each corresponding stage; blue: Hoechst nucleic acid staining). The frequency at which a given area displayed GFP signal (infection index) increased from E17 (n = 18 embryonic brains from two pregnant rats) to E20 (n = 46 embryonic brains from five pregnant rats) and P1 (n = 38 neonatal brains from four pregnant rats). Similarly, the absolute number of GFP^+^, infected cells as counted in a subset of the aforementioned brains (n = 11 brains at E17; n = 11 brains at E20; n = 13 brains at P1) (Table C in S1 File) increased significantly (Kruskall-Wallis test followed by Dunn's post-hoc test) from E17 to P1 in the corresponding brain areas of interest. For each developmental stage and brain area analyzed, each dot represents the number of GFP+ cells per ROI in a given brain. ROI (387x387 μm^2^): region of interest. OB: olfactory bulb. LV: lateral ventricle. CP: choroid plexi. Th: thalamic area of the third ventricle. Hyp: hypothalamic area of the third ventricle. Areas are shown upon a rostrocaudal axis, from top to bottom. White bars: 1 mm; yellow bar: 200 μm; red bar: 40 μm. ns: non significant; *: p < 0.05; **: p < 0.01; ****: p<0.0001.

The kinetics of infection by GFP-expressing recombinant RCMV within the fetal brain was analyzed after icv injection at E15 by detection of green fluorescent protein (GFP) in the infected cells (Fig 1; Figure A in S1 File). GFP detection was indeed more effective in assessing the presence of active RCMV within the cells, than antibodies against viral protein R44 (Figure B in S1 File). As expected and also recently shown in the case of its murine CMV counterpart [9], RCMV was increasingly detected in the infected brains after icv injection. At E16 (*i*.*e*. 24h after icv injection), no GFP was detected (n = 8 embryonic brains) (data not shown). At E17, only a few GFP-positive (GFP^+^) cells were counted (n = 11 embryonic brains), and GFP signal was most frequently detected in the choroid plexi and in the thalamic and hypothalamic areas (n = 18 embryonic brains) (Fig 1; Table C in S1 File). At E20, more GFP^+^ cells were counted (n = 11 embryonic brains) and the infection expanded to other brain areas (olfactory bulbs, lateral ventricles) (n = 46 embryonic brains) (Fig 1; Table C in S1 File). At P1, there was a dramatic increase in the number of infected cells as compared to E17 (n = 13 brains), and in the frequency at which GFP could be detected in a given brain area (n = 38 brains); this included the choroid plexi, the ventricular and subventricular zones and notably the thalamic and hypothalamic areas, and the olfactory bulbs (Fig 1; Table C in S1 File). CMV was also consistently detected in the meninges, the cerebellum, the inner ear and the hippocampal area (data not shown). In line with the increasing number of infected cells and with the expansion of CMV infection to more brain areas, GFP and R44 gene expressions also increased from E16 to P1 (Figure B in S1 File).

### Most RCMV-infected cells are of the myeloid or lymphoid lineages

**Fig 2:**
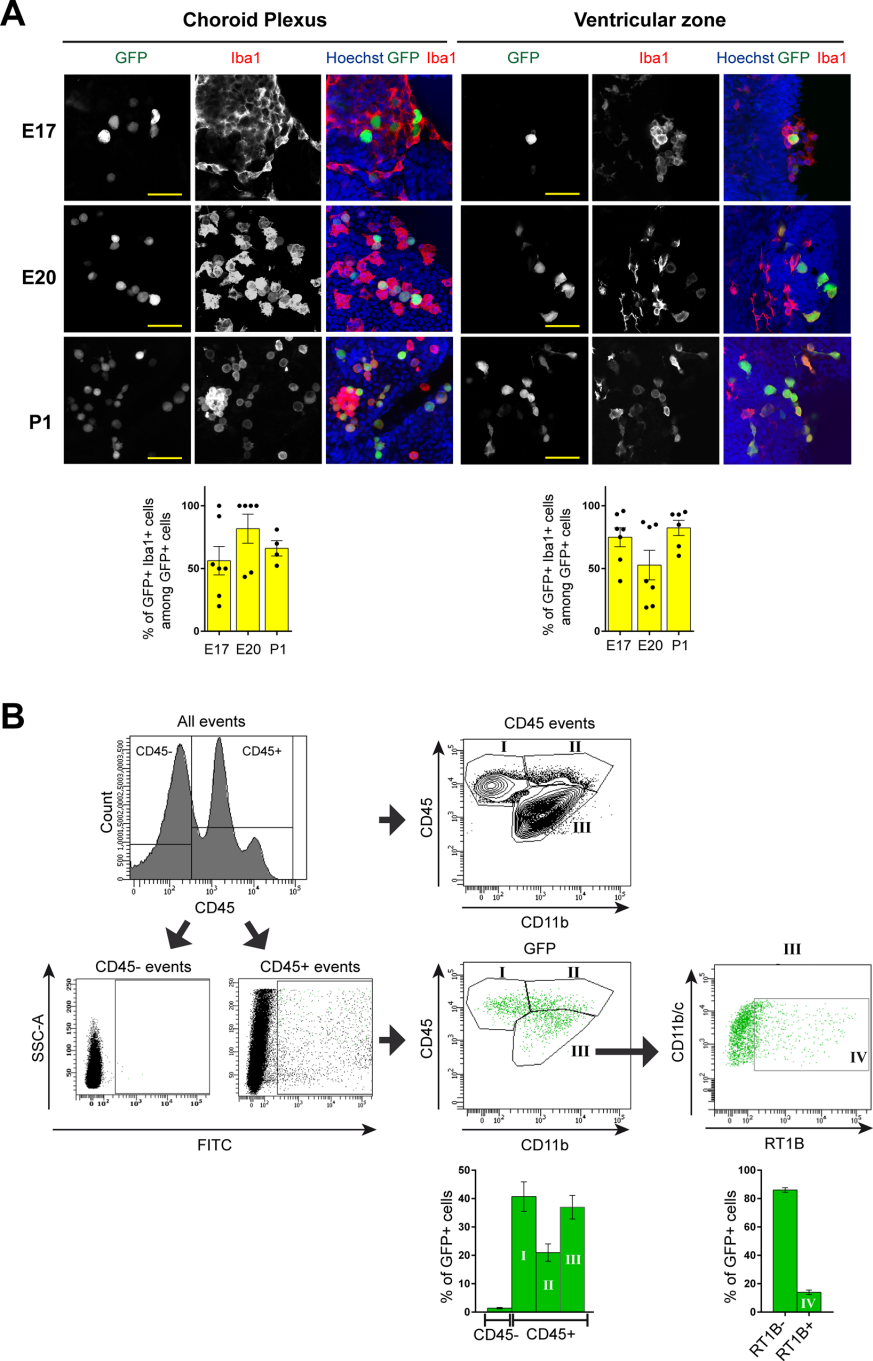
Early prominent infection of microglial cells and brain macrophages following rat CMV (RCMV) administration *in utero*. (A) Immunocytochemistry experiments (Top) The choroid plexus and the ventricular area of the infected brains were analyzed using confocal microscopy at various developmental time points (E17, E20, P1) after RCMV (GFP, green) infection at E15. Coronal slices were stained with antibodies against microglia/macrophages marker Iba1 (red). Nuclei were stained with Hoechst dye (blue). Infected cells expressing GFP (green) frequently showed expression of Iba1 (red) (Bottom) Cell counting (see also Table D in S1 File) demonstrating that a large proportion of infected (GFP^+^) cells in the choroid plexus (left) (n = 102 cells at E17; 169 cells at E20; 741 cells at P1) and in the ventricular zone (right) (n = 31 cells at E17; 100 cells at E20; 563 cells at P1) are microglia/macrophages (GFP^+^ Iba1^+^, yellow). For each developmental stage and brain area analyzed, each dot represents the proportion of GFP+ Iba1+ cells amongst all GFP+ cells per ROI in a given brain. ROI (387x387 μm^2^): region of interest. Yellow bar: 40 μm. (B) Characterization of RCMV-infected leukocytes by flow cytometry. Leukocytes were isolated from the infected brains at P1 and then gated for CD45 and GFP expressions. GFP^+^ CD45^+^ cells (top) were further characterized for CD11b (bottom, left) marker. Histograms (bottom, right) show the relative distribution of CD45^+^ CD11b- cells (fraction I: B cells, T cells and non-B non-T cells), of CD45^high^ CD11b^+^ cells (fraction II: infiltrating myeloid cells), and of CD45^low/int^ CD11b^+^ cells (fraction III: microglia), amongst all infected leukocytes. Cells from fraction III (microglia) were further characterized according to RT1B expression status, allowing to determine the proportion of infected, activated microglia (fraction IV). Values are reported as mean ± SEM of 18 RCMV infected brains.

The infected cell types were first characterized by immunohistochemistry experiments at E17 and P1 developmental stages. The majority of infected (GFP^+^) cells in the choroid plexi and in the lateral ventricular areas were Iba1^+^, CD45^+^ immune cells likely corresponding to microglia/brain macrophages (Fig 2A; Figure C in S1 File; Table D in S1 File). Other GFP^+^ cell types were occasionally detected (Figure D in S1 File). This included cells expressing NG2, a marker of pericytes and oligodendrocytes precursors, and cells expressing S100β, a marker of ependymal cells and of astrocytes. RCMV was also detected in some aquaporin-1 expressing epithelial cells of the choroid plexi. No GFP signal was detected in NeuN^+^ mature neuronal cells (data not shown). Although nestin^+^ radial glial progenitor cells did not show GFP expression, infected Iba1^+^ cells were frequently found in close contact with radial glial cells (Figure E in S1 File). Interestingly, the structure of the radial glia lying in the immediate vicinity of such periventricular GFP^+^, Iba1^+^ infected cells was altered while it appeared unaltered for radial glia located more distal to the site of infection (Figure E in S1 File). No broad alteration in the cortical lamination of the dorsal telencephalon was significantly detected at P1 with markers of deep (Foxp2), intermediate (Ctip2) and upper (Cux1) cortical layers, as compared with control (MEM-injected) situation (data not shown). As mentioned above, only a few infected cells were detected at E17, and very rarely in the ventricular zone of the dorsal telencephalon (Fig 1); as E17 nearly corresponds to the end of the period when radial glia-guided neuronal migration occurs (E12-E18), lamination may only be altered unfrequently and at very localized areas in the present model. Also, such localized anomalies of cortical lamination caused by focal alterations of radial neuronal migration may well have escaped detection.

To further characterize the infected cells, flow cytometry analysis was performed at P1 (n = 18 brains) on CD45^+^ hematopoietic cells from RCMV infected brains. Hematopoietic cells were separated in three populations according to their CD11b and CD45 expression profile to differentiate between brain resident microglial cells from other infiltrating myelo-monocytic and lymphocytic cell populations, as previously reported [13](Fig 2B). Among these subsets, RCMV infected cells were identified by analyzing the expression of GFP. Of note, the small number of GFP^+^ cells at E17 prevented reliable flow cytometry analysis of infected cells at that stage. Amongst the GFP^+^, CD45^+^ cells, 40.7% ± 5.2 were found in fraction I of CD45^high^ CD11b- cells entailing B and T-cells as well as other type of leukocytes. Another 20.9% ± 3.0 of infected leukocytes were CD45^high^, CD11b^+^ (fraction II) corresponding to myeloid lineage cells (monocytes/macrophages, dendritic cells (DC) and granulocytes). The remaining CD45^low/int^, CD11b^+^ cells (36.9% ± 4.1) found in fraction III entailed resident microglial cells. Only a minority of such infected microglial cells were in an activated (RT1B+) state (fraction IV; 15.2% ± 2) (Fig 2B); down-regulation of RT1B MHC classII antigen expression in RCMV-infected cells has indeed been reported previously [10].

### Areas of RCMV infection show an early increase in microglia/brain macrophages activation

**Fig 3:**
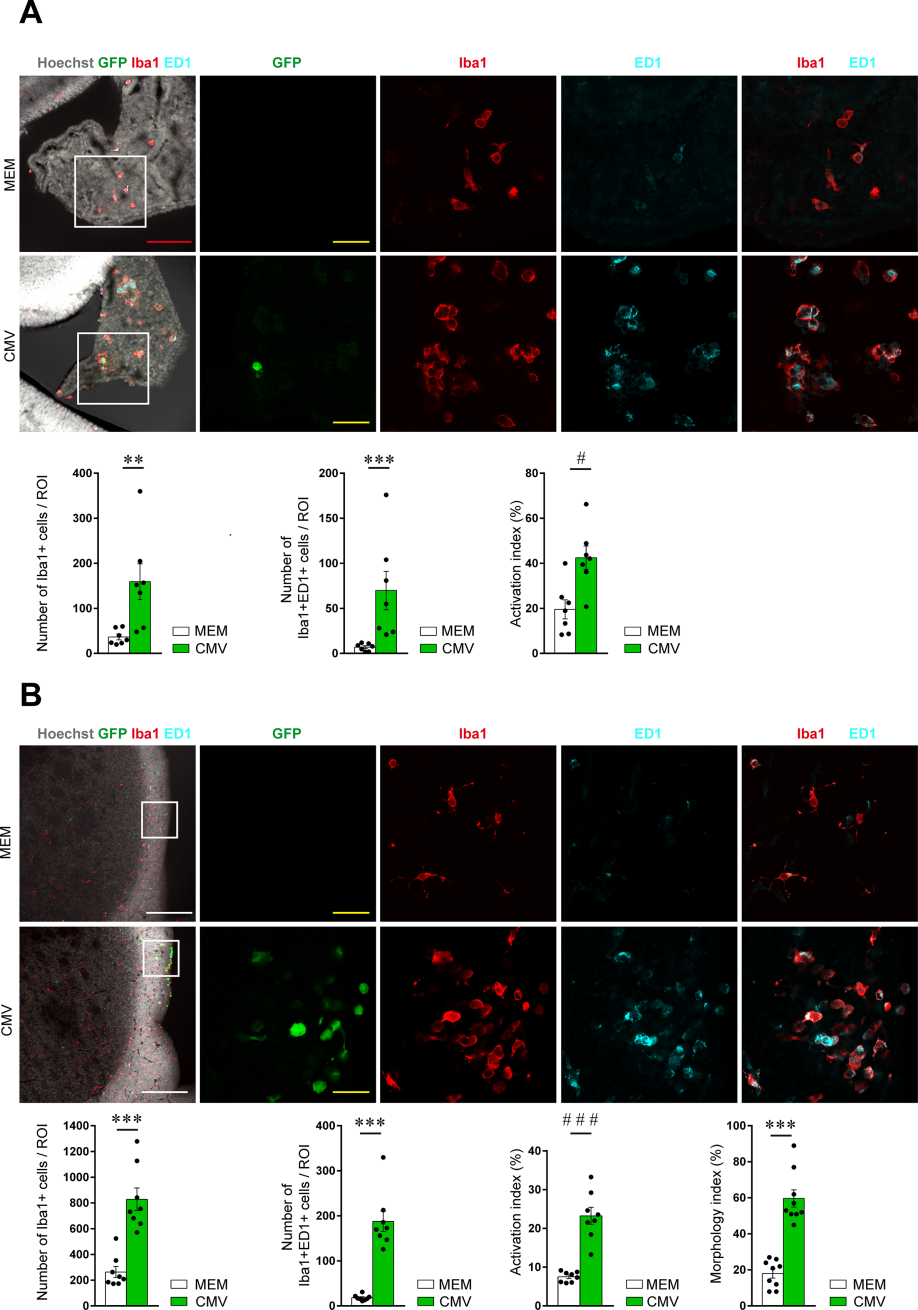
Early activation of microglia/brain macrophages in rat CMV-infected areas of the developing brain. The activation status of macrophages of the choroid plexus (A) and of microglia/macrophages of lateral ventricles (B) were assessed by immunohistological experiments on coronal sections observed by confocal microscopy at E17 (n = 7 brains) and at P1 (n = 8 brains), respectively. Increased number of Iba1^+^ cells (red) and of their activation status (Cd68/Ed1^+^, cyan) was demonstrated in CMV-infected areas (see also Table E in S1 File). Activation index was calculated as the ratio of the number of Iba1^+^, Ed1^+^ activated cells versus total number of Iba1^+^ cells. Morphology index was calculated as the ratio of the number of amoeboid activated, Iba1^+^ cells versus total number of Iba1^+^ cells. Each dot represents a single brain. MEM: minimum essential medium (control condition). CMV: cytomegalovirus. ROI: region of interest (387x387 μm^2^). Green fluorescent protein (GFP, green) expression indicates the CMV-infected cells. White bar: 200 μm; red bar: 100 μm, yellow bar: 30 μm. Mann Whitney test, two tailed, with Bonferroni correction wherever appropriate; **: p < 0.01***: p < 0.001; #: p < 0.025; ###: p < 0.0005.

Microglial cells/brain macrophages can rapidly shift out of a quiescent state to become activated upon various insults of the central nervous system (CNS), such as infections, stroke, or chemical exposure. In our model, the number of Iba1^+^ cells and the phagocytic activity of those cells, as estimated upon expression of the phagocytic activation marker Ed1/Cd68 [14], [15], [16], [17] both increased significantly in the RCMV-infected areas of the developing brain at E17 (Iba1^+^ cells: 159.1±39.6/ROI; Iba1^+^Cd68^+^ cells: 69.9±21.3/ROI) (Fig 3A; Table E in S1 File) and at P1 (Iba1^+^ cells: 828.9±87.7/ROI; Iba1^+^Cd68^+^ cells: 187.6±22.3/ROI) (Fig 3B; Table E in S1 File), as compared with control brains at E17 (Iba1^+^ cells: 36.4±6.3/ROI; Iba1^+^Cd68^+^ cells: 7.0±1.5/ROI) and at P1 (Iba1^+^ cells: 263.8±42.8/ROI; Iba1^+^Cd68^+^ cells: 18.3±2.2/ROI). Consistently, RCMV infection was accompanied locally by a dramatic change in the morphology of Iba1^+^ cells, with a significant shift from the ramified phenotype of quiescent cells to the classical amoeboid morphology of activated cells (proportion of amoeboid cells at P1: 60.9%±5.3 in CMV condition; 16.8±2.6 in control condition) (Fig 3B; Table E in S1 File).

### RCMV infection of the developing brain leads to chemokine dysexpression and to an early infiltration by myelomonocytes and by lymphocytes

CMV interferes with the host chemokine/receptor system by dysregulating the expression of cellular chemokines, and by encoding endogenous viral CMV chemokines [18]. Such host and viral chemokines might have promoted infiltration of the brain by monocytes-derived macrophages and by lymphocytes. To determine whether and when the expression of various cytokines and chemokines would be altered upon RCMV infection of the developing brain, qRT-PCR experiments were performed at various developmental stages (Figure F in S1 File). In addition to the early detection (as from E16) of viral chemokine r129 gene expression, we detected an early, significant after correction for multiple testing, and sometimes dramatic upregulation of several genes encoding: host cytokine IL-10 (at P1: fold change 4.57±1.03; p = 0.0006); and pro-inflammatory chemokines CCL2 (at E17: fold change 2.40±0.14; p = 0.0003), CCL3 (at E17: fold change 1.63±0.10; p = 0.0047), CCL4 (at E17: fold change 5.12±0.76; p = 0.0003; at P1: fold change 6.50±1.59; p = 0.0002), CCL7 (at E17: fold change 2.88±0.43; p = 0.0011; at P1: fold change 4.98±1.39; p = 0.0002) and CCL12 (at E17: fold change 27.52±8.89; p = 0.0002; at P1: fold change 41.40±14.38; p = 0.0002).

**Fig 4:**
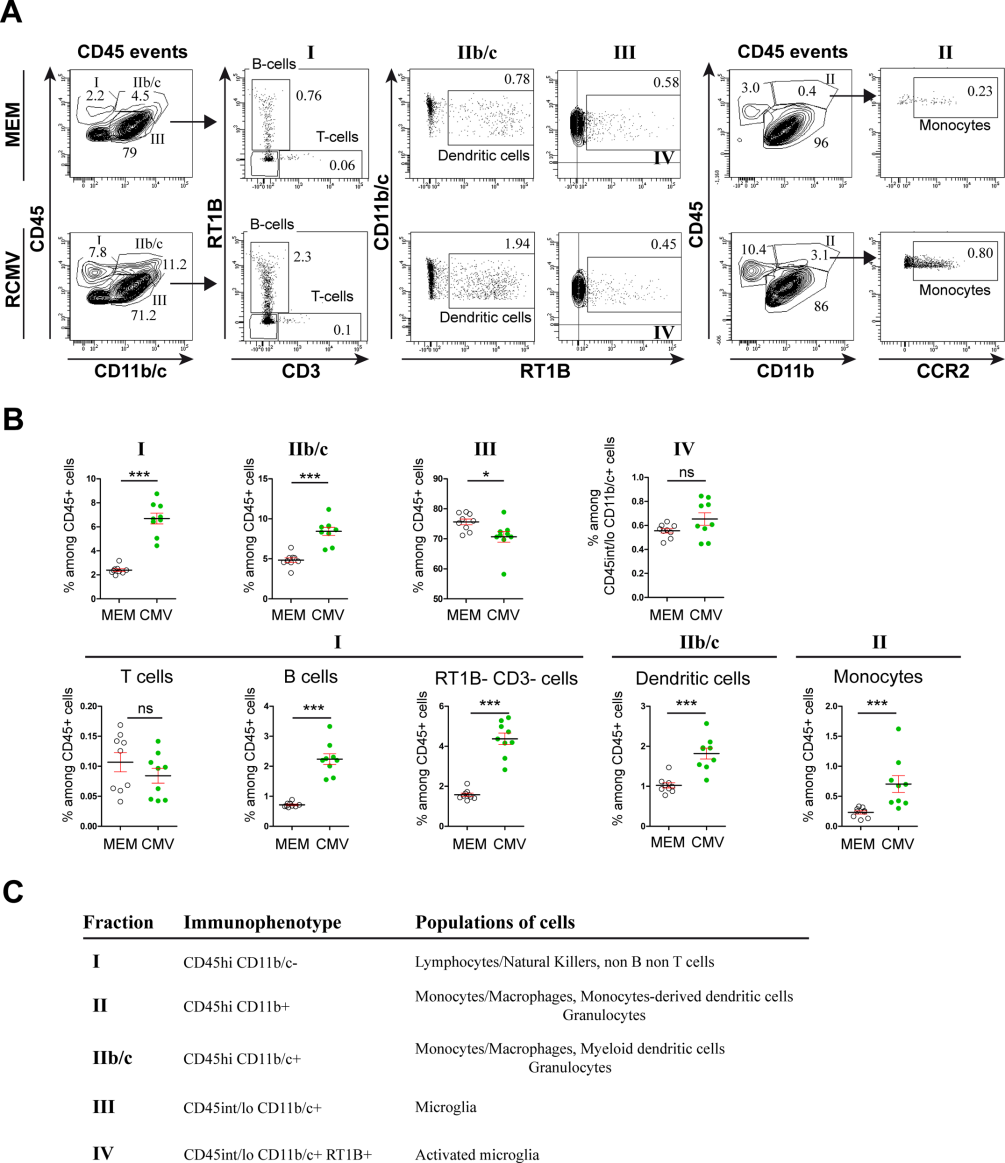
Flow cytometry analysis of leukocytes collected at E17 from RCMV-infected brains. Total leukocytes (CD45 events) were gated for CD45 and either Cd11b/c or CD11b expressions and further characterized for CD3 and RT1B or for CCR2 expressions, respectively (A) Representative flow cytometry plots in control (MEM-injected) brains and (RCMV) brains. (B) (top) The distribution of fractions I, IIb/c and III as defined according to CD45/CD11b/c expression status, was significantly different in CMV-infected brains as compared with control (MEM) brains at E17. Cells from fraction III (microglia) were further characterized in either conditions according to RT1B expression status, allowing to determine the proportion of activated microglia (fraction IV). No difference in the proportion of activated microglia was observed in the infected brains at E17 (bottom) Cells from fraction I were further characterized in either conditions according to CD3 and RT1B expression status, allowing to distinguish between T cells, B cells and non-B non-Tcells. The proportions of B cells and of non-B non-T cells increased significantly in CMV-infected brains at E17. Dendritic cells from fraction IIb/c were characterized in either conditions according to RT1B expression status whereas monocytes were characterized in either conditions according to CD45/CD11b (corresponding to fraction II) and CCR2 expression status. The proportions of dendritic cells and of monocytes increased significantly in CMV-infected brains at E17. All analyzes were performed in either of control (MEM) or infected (RCMV) conditions using nine pools of four embryonic brains each. Values are means ± SEM. The statistical significance of the observed variation in frequency in each cell population is indicated. Mann Whitney test, two tailed; ns: non significant; *: p < 0.05; ***: p < 0.001 (C) Fractions of cells as defined according to their respective immunophenotypes and the cell populations they correspond to, are indicated.

**Fig 5:**
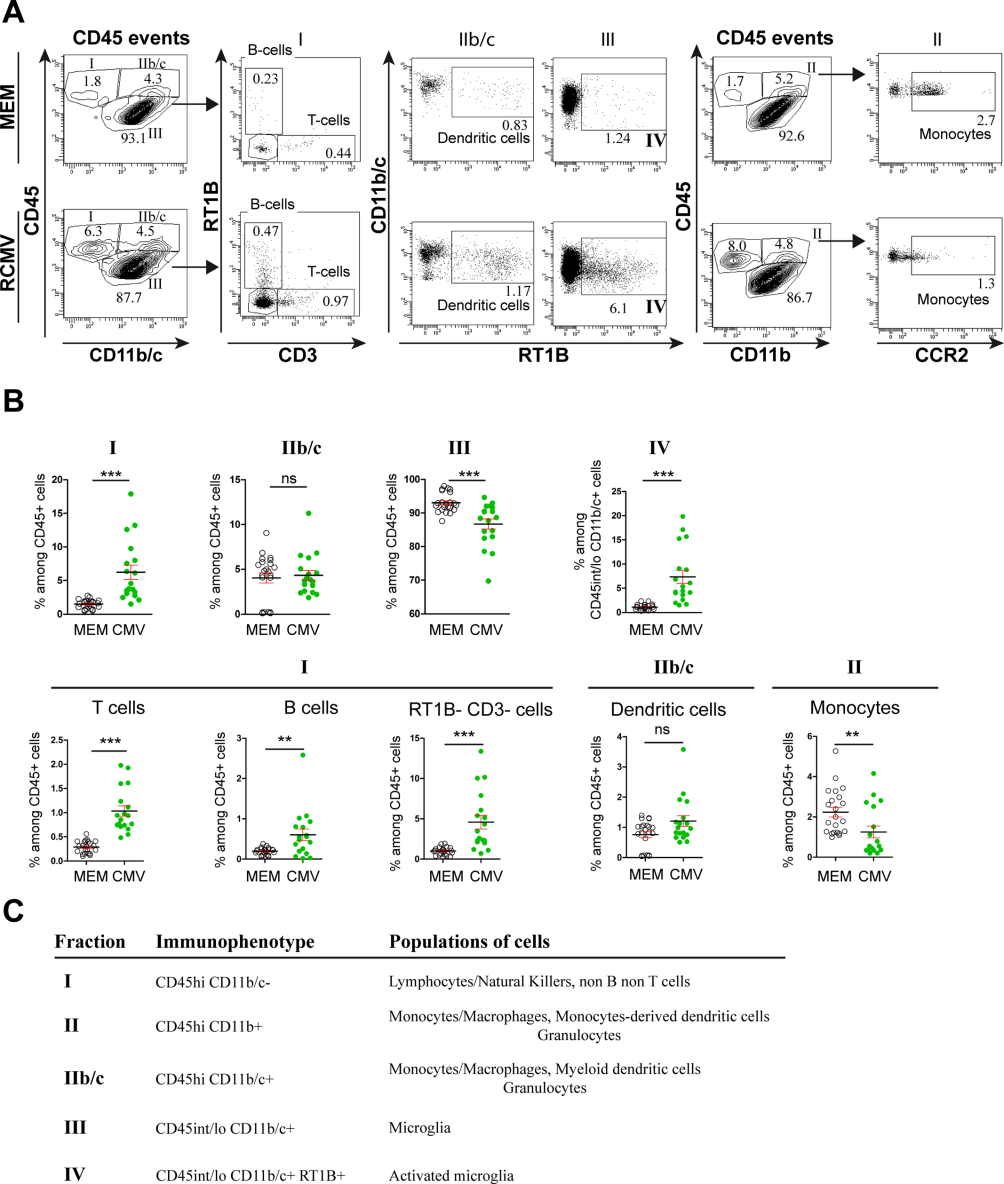
Flow cytometry analysis of leukocytes collected at P1 from RCMV-infected brains. Total leukocytes (CD45 events) were gated for CD45 and either Cd11b/c or CD11b expressions and further characterized for CD3 and RT1B or for CCR2 expressions, respectively (A) Representative flow cytometry plots in control (MEM-injected) brains and (RCMV) brains. (B) (top) The distribution of fractions I and III, but not IIb/c, as defined according to CD45/CD11b/c expression status, was significantly different in CMV-infected brains as compared with control (MEM) brains at P1. Cells from fraction III (microglia) were further characterized in either conditions according to RT1B expression status, allowing to determine the proportion of activated microglia (fraction IV). The proportion of activated microglia increased significantly in the infected brains at P1 (bottom) Cells from fraction I were further characterized in either conditions according to CD3 and RT1B expression status, allowing to distinguish between T cells, B cells and non-B non-T cells. The proportions of T cells, B cells and non-B non-T cells increased significantly in CMV-infected brains at P1. Dendritic cells from fraction IIb/c were characterized in either conditions according to RT1B expression status whereas monocytes were characterized in either conditions according to CD45/CD11b (corresponding to fraction II) and CCR2 expression status. The proportions of monocytes increased significantly in CMV-infected brains at P1. No difference in the proportion of dendritic cells was observed in the infected brains at P1. All analyzes were performed using 18 (RCMV) and 22 (control condition) brains, respectively. Values are means ± SEM. The statistical significance of the observed variation in frequency in each cell population is indicated. Mann Whitney test, two tailed; ns: non significant; **: p < 0.01; ***: p < 0.001 (C) Fractions of cells as defined according to their respective immunophenotypes and the cell populations they correspond to, are indicated.

Consistent with this dysregulation of pro-inflammatory chemokines, flow cytometry analysis targeted towards GFP^+^ infected leukocytes in RCMV-infected brains had already indicated that a subset of those cells had peripheral origin (Fig 2B). Mononuclear cells were purified from whole infected brains at E17 and P1 regardless of their possible infected status and analyzed further by flow cytometry (Fig 4). Hematopoietic cells were separated according to their CD45 and CD11b/c or CD11b expression status. The lineage of the cells found in the different fractions was further precised using additional set of markers. In fraction I, B cells (RT1B^+^ CD3-), T cells (RT1B- CD3^+^) and non-B non-T cells (RT1B- CD3-) were resolved. Infiltrating myeloid cells were further separated into dendritic cells (DC, RT1B^+^ CD11b/c^+^) and monocytes (CCR2^+^ CD11b^+^). Fourty eight hours after RCMV injection and as compared with control brains (n = nine pools of four embryonic brains each in either condition), the proportion of B cells (CMV: 2.24%±0.18; MEM: 0.71%±0.03; p < 0.0001) and non-B non-T cells (CMV: 4.37%±0.28; MEM: 1.58%±0.08; p < 0.0001) as well as of monocytes (CMV: 0.70%±0.14; MEM: 0.23%±0.03; p < 0.0002) and DCs (CMV: 1.82%±0.14; MEM: 1.02%±0.06; p < 0.0001) significantly increased in the infected brains at E17. As a corollary of such increases in fractions I and II, the relative proportion of the microglia cells (fraction III) significantly decreased (CMV: 70.69%±1.80; MEM: 75.65%±0.95; p = 0.0188). In contrast with the local increase in the activation status of microglia seen at E17 in the infected areas by immunohistochemistry, microglial activation status (fraction IV) remained unchanged in whole E17 brains as identify by the absence of RT1B upregulation [19] (fraction IV) (CMV: 0.65%±0.05; MEM: 0.55%±0.02; p = 0.19). At P1 (Fig 5), the proportion of all three cell populations in fraction I (T, B and non-B non-T cells) increased significantly in RCMV-infected brains as compared with control brains (T-cells: CMV: 1.03%±0.11; MEM: 0.29%±0.02; p < 0.0001. B-cells: CMV: 0.60%±0.14; MEM: 0.20%±0.02; p = 0.0064. non-B non-T cells: CMV: 4.60%±0.85; MEM: 1.00%±0.10; p < 0.0001). In fraction IIb/c the proportion of DC (RT1B+ CD11b/c+) remained unchanged (CMV: 1.21%±0.18; MEM: 0.76%±0.10; p = 0.19) whereas in fraction II, the proportion of monocyte (CCR2^+^) was significantly reduced (CMV: 1.25%±0.29; MEM: 2.24%±0.24; p = 0.0033). In addition, the proportion of microglia decreased significantly as compared with control brains (CMV: 86.69%±1.52; MEM: 93.05%±0.61; p < 0.0001); however and in contrast with E17, an overall activation of microglia (fraction IV) was significantly observed at P1 (CMV: 7.35%±1.36; MEM: 1.10%±0.12; p < 0.0001), likely reflecting the dramatic increase in the number of CMV-infected brain areas (Fig 1) and the increased expression of proinflammatory cytokines (Figure F in S1 File) seen from E17 to P1. As compared with E17, at P1 the infected brains showed a significant increase in the proportions of activated microglia (p < 0.0001) and of T-cells (p < 0.0001), and a significant decrease in the proportion of monocytes (p < 0.0001) (Figure G in S1 File).

## Discussion

Because of the species-specificity of CMVs, animal models of congenital CMV infection of the developing brain have been designed in order to decipher the underlying pathogenesis. Hence, various antenatal and neonatal rodent models with intracranial, intraperitoneal or intraplacental CMV inoculation have been used, particularly in the mouse. In the present study, we have designed and developed a model of RCMV infection *in utero*, where the virus is being inoculated directly into the cerebral ventricles of the embryos at a specific developmental time-point. Whereas the use of neonatal models of brain CMV infection has brought important insights into the possible pathogenic mechanisms, antenatal models that also take into account *in utero* events are needed. As an example, the period of expansion of RCMV dissemination seen in the rat model reported here between E20 and P1, paralleled the burst of microglial dissemination occurring as from E19-E20 in the normal developing rat brain [20]. Generally, events preceding and surrounding birth might be critical in modulating the consequences of intrauterine insults [21]. Intraventricular mode of administration as used here and elsewhere [22, 23]. obviously does not recapitulate the time course of mother-to-fetus and periphery-to-brain transmission of the human pathology. However it is very appropriate for investigating the early events that follow infection of the developing CNS. It also limits the huge variability of other complicated events taking place in the placenta and in peripheral tissue prior to brain infection. Despite its relative simplicity and inherent limitations, our model shared several aspects in common with human congenital CMV infection, including focal infection of the periventricular areas surrounding the lateral and third ventricles. Notably the detection of RCMV in the periventricular zones recalled the periventricular localization of calcifications classically seen in human congenital CMV ventriculoencephalitis. Other convergent localizations with the human disease were the frequent infection of the inner ear, of the olfactory bulbs and of the hippocampal area, as seen in infected fetuses [24, 25]. Periventricular areas and olfactory bulbs, but also choroid plexi, are major sites of early infection in neonatal murine models of brain infection by other types of viruses such as coxsackievirus B3 [26] and lymphocytic choriomeningitis virus [27]. Generally choroid plexi, which represent a key interface between blood and cerebrospinal fluid, may play multiple pathogenic roles, either as entry and dissemination sites of pathogens into the CNS, or as sources of cytokines and chemokines production and of recruitment of immune cells. The role of choroid plexi in the pathogenesis of meningitis caused by coxsackievirus B3 has indeed recently been emphasized [28].

At the cellular level, the frequent and early detection of RCMV in microglial cells and in brain macrophages as shown here, confirmed previous findings indicating that such brain immune cells are infected not only by human CMV in fetuses [25], but also by MCMV in murine models of *in utero* intraplacental infection [9] or of neonatal intracerebral infection [29]. Microglia might well be the primary target of CMV upon brain infection of the developing brain; as a matter of fact, microglia express several different integrins including beta-1 integrin [30], and it was recently shown that beta-1 integrin expressing cells were the primary infected cells in a mouse model of neonatal infection [23]. In contrast with murine models, neural progenitor cells (NPCs) were not targeted here, at least from two to seven days after RCMV inoculation. This might reflect peculiar attenuated neural tropism of the RCMV strain used here. Alternatively, NPCs from various species or even from various strains of rat might show strikingly different susceptibility to CMV entry or replication. As mentioned above, the frequent, immediate nearness of nestin NPCs with infected GFP microglial cells could sometimes even have been mistaken as an actual colocalization of the two molecules, which could be excluded only with closer and 3D systematic examinations. Indeed, microglial cells closely adjacent to nestin cells have also been detected in murine neonatal pups [31]. Nevertheless the corresponding local alterations of the radial glia structure found in our model indicated that NPCs were indirectly altered by the presence of infected cells in their immediate neighborhood.

CMV infection was associated with an early activation of microglia at the site of active CMV infection, with an infiltration by peripheral immune cells, and with an early increase in chemokine gene expression. There has indeed been increasing and convergent evidence in various murine models for the important role of inflammation associated with innate and adaptive immune responses in the pathophysiology of congenital CMV infection of the brain [32] [5, 9, 33, 34],[7]. Also, histopathological studies on human brains from autopsied fetuses detected frequent microglial nodules [25]. Here, infiltrating myelomonocytes and lymphocytes were detected at E17, whereas at P1, lymphocytes were detected as the main infitrating population. Induction by human CMV of monocyte migration and differentiation into macrophages has already been demonstrated [35, 36]. Also, it was shown that following intraperitoneal injection of CMV in newborn mice, monocytes/macrophages were amongst the most abundant early infitrating cells, whereas lymphocytes represented the majority of cells thereafter [7]. Consistently, several genes encoding monocytes and lymphocytes chemoattractants were upregulated early upon CMV inoculation in our model. Induction of various cytokines and chemokines by CMV, notably in microglia, has already been demonstrated [6]. In our study, this included viral protein r129, which was recently shown to attract lymphocytes and macrophages [18], and cellular chemokines CCL2, CCL3, CCL4, CCL7 and CCL12. Chemoattractant protein CCL2 expression by microglial cells promotes the infiltration of monocytes into the brain [37], whereas CCL12 expression in the choroid plexus was shown to play a crucial attracting role on peripheral cells of the myeloid lineage following coxsackievirus infection of the neonatal brain [38]. Interestingly also, upregulation of the anti-inflammatory IL-10 cytokine gene as seen here at P1 might combat against the production of proinflammatory compounds by activated microglia [39].

The role of NK cells and of T cells in controlling CMV infection has already been suggested [40]. In a model of intracerebroventricular CMV infection of mice aged 6–8 weeks, it was also shown that a subset of infiltrating regulatory B cells controls T lymphocytes and microglial responses [8]. Generally the early recruitment of peripheral immune cells, including monocytes and DCs, and the increased activation of microglial cells are likely to play important roles in the pathogenesis of congenital CMV infections of the brain. There is evidence to suggest that infiltrating monocytes have the capacity to give rise to microglial cells in some models of CNS infection [41]. In our model, the infiltration by monocytes and DC occurred in E17-infected brains but was less prevalent in P1 infected brains as compared to control brains. As the reduction of the monocyte pool was concomitant with an increase of fraction IV cells at P1 stage, it could be inferred that infiltrating monocytes differentiated into activated microglia in the course of congenital CMV infection. Here, evidence for microglial activation, which is a complicated and heterogeneous process [42], was obtained by a combination of immunohistochemistry, morphological and flow cytometry analyzes. Whereas early microglial activation within the sites of productive infection is likely to regulate CMV infection, it might also have detrimental effects. Notably the role of microglia in shaping the brain during development, including synaptogenesis, synaptic pruning, or neocortical interneuron positioning, and in modulating synaptic plasticity and neuronal networks, is increasingly recognized [43]. The direct targeting of microglial cells by CMV and the modification in the activated state of microglia *in utero* might well have postnatal impact on brain functioning and pathology [44], strongly influencing, or at least interfering with, the outcome of congenital CMV infection and the severity of the related neurological manifestations. As an example, long-lasting effects of transient activation of microglia on adult neurogenesis in the olfactory bulb have been reported [45]. Generally disturbance in microglia functioning likely participates in various disorders of the CNS [46]. In the present rat model, prominent interaction of CMV with microglia and macrophages was shown in the developing brain; although our model inherently does not take into account all aspects of congenital infections, including transplacental infection, it represents a very useful and quite unique tool to decipher the specific pathogenic involvement of those cells in the brain, which are likely to contribute the corresponding human disease. In conclusion, our model recapitulated several key features of the corresponding human congenital disease as well as of the previously reported murine models. Our findings suggest that the early alteration in the activation state of microglial cells, the infiltration by peripheral leukocytes, and the transcriptional upregulation of various chemokines, might play important roles in the initial stages following CMV infection of the developing brain *in utero*. Ongoing and future studies will help in determining which important role those immune responses actually play, whether they are favorable or detrimental to CMV dissemination within the developing brain, and how they would cause or influence the possible postnatal emergence of various cerebral disorders.

## Supporting Information

S1 File Figure A. Morphological Analysis of control brains at various developmental stages. Figure B. Detection of RCMV-infected cells with GFP and with R44 antibodies, and of *GFP* and *R44* gene expressions. Figure C. Colocalization of RCMV-infected brain cells with CD45 and Iba1 markers at P1. Figure D. Colocalizations of RCMV-infected brain cells with NG2, S100beta and AQP1 markers at P1. Figure E. Lack of infection and altered morphology of radial glial cells in RCMV-infected brains. Figure F. Dysexpression of various cytokines and chemokines genes upon RCMV infection of the developing brain. Figure G. Evolution of cell distribution from E17 to P1 in CMV-infected brains by flow cytometry analysis. Table A. List of antibodies used in immunohistochemistry and flow cytometry. Table B. List of primer sequences. Table C. Absolute numbers of GFP+ cells as determined by cell counting in various brain areas of interest at E17, E20 and P1 (see also Fig 1). Table D. Absolute numbers of GFP+ cells and of GFP+ Iba1+ cells as determined by cell counting in the choroid plexi and in the lateral ventricles of RCMV-infected brains at E17, E20 and P1 (see also Fig 2A). Table E. Absolute numbers of Iba1+ cells and of Iba1+ Ed1+ cells as determined by cell counting at E17 (see also Fig 3A) and at P1 (see also Fig 3B).
(PDF)
