# Supplementary material for: Cytomegalovirus Infection of the Rat Developing Brain In Utero Prominently Targets Immune Cells and Promotes Early Microglial Activation
Source: PLoS One. 2016 Jul 29;11(7):e0160176. doi: 10.1371/journal.pone.0160176 (PMC4966896; doi:10.1371/journal.pone.0160176)
Supplement: S1 File — Figure A. Morphological Analysis of control brains at various developmental stages. Figure B. Detection of RCMV-infected cells with GFP and with R44 antibodies, and of GFP and R44 gene expressions. Figure C. Colocalization of RCMV-infected brain cells with CD45 and Iba1 markers at P1. Figure D. Colocalizations of RCMV-infected brain cells with NG2, S100beta and AQP1 markers at P1. Figure E. Lack of infection and altered morphology of radial glial cells in RCMV-infected brains. Figure F. Dysexpression of various cytokines and chemokines genes upon RCMV infection of the developing brain. Figure G. Evolution of cell distribution from E17 to P1 in CMV-infected brains by flow cytometry analysis. Table A. List of antibodies used in immunohistochemistry and flow cytometry. Table B. List of primer sequences. Table C. Absolute numbers of GFP+ cells as determined by cell counting in various brain areas of interest at E17, E20 and P1 (see also Fig 1). Table D. Absolute numbers of GFP+ cells and of GFP+ Iba1+ cells as determined by cell counting in the choroid plexi and in the lateral ventricles of RCMV-infected brains at E17, E20 and P1 (see also Fig 2A). Table E. Absolute numbers of Iba1+ cells and of Iba1+ Ed1+ cells as determined by cell counting at E17 (see also Fig 3A) and at P1 (see also Fig 3B). (PDF) [file pone.0160176.s001.pdf]

S1 file - Supporting information online.

Figure A. Morphological Analysis of control brains at various developmental stages.

MEM (the vehicle) was injected intraventricularly at E15. Coronal sections of brain areas were observed at various developmental time points (E17, E20 and P1; n = 18, 9 and 31 brains respectively) using confocal microscopy (blue: Hoechst nucleic acid staining). No GFP signal was ever observed. Areas are shown upon a rostrocaudal axis, from top to bottom. White bar: 100 μm; yellow bar: 30 μm.

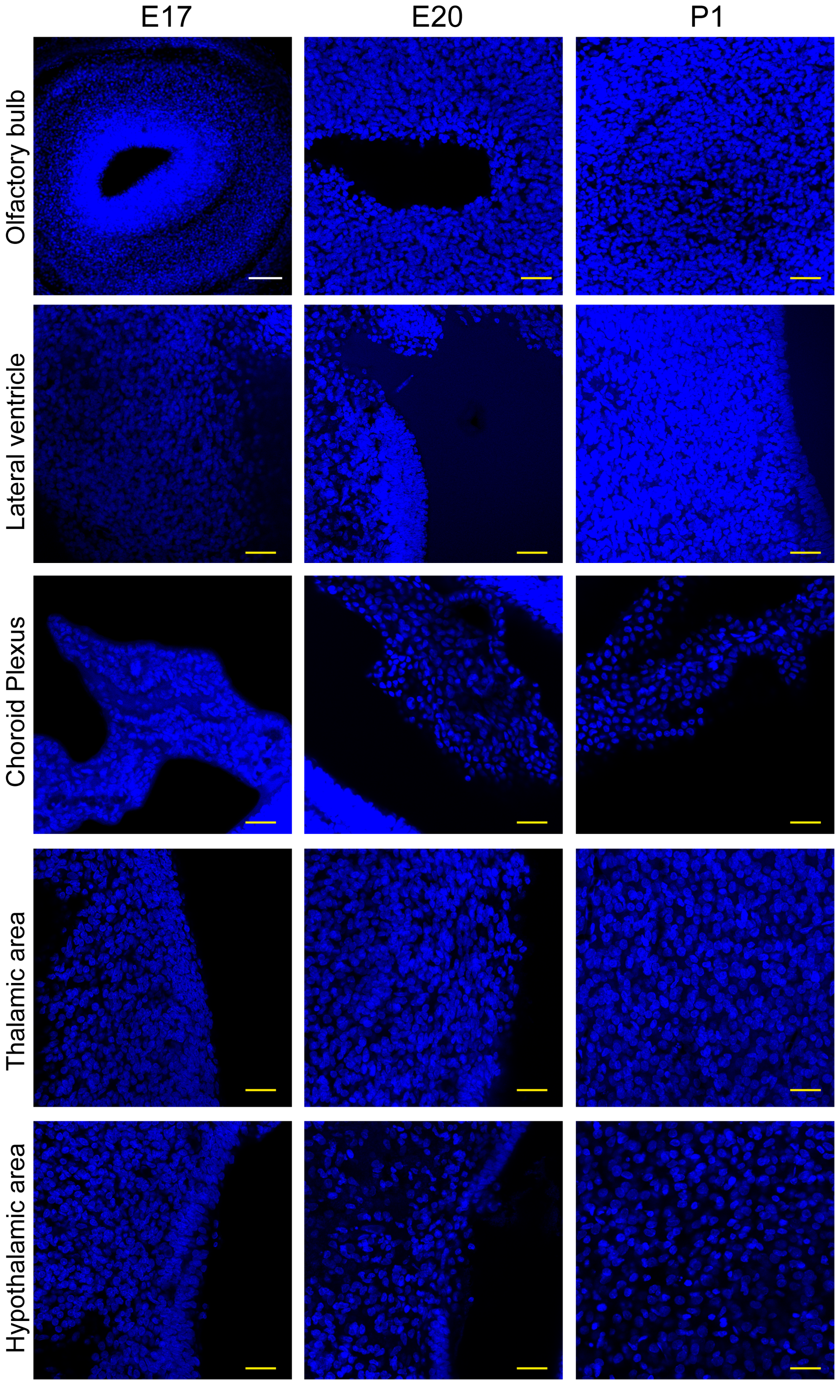

Figure B. Detection of RCMV-infected cells with GFP and with R44 antibodies, and of *GFP* and *R44* gene expressions.

(A) RCMV-infected cells were detected at E17 (choroid plexi) and at P1 (lateral ventricles). Cells were also detected with antibodies against R44 early/late viral protein (red). Note that GFP was more effective in detecting RCMV-infected cells. Nuclei staining: Hoechst (blue). Yellow bar: 30 μm (B) qRT-PCR experiments demonstrated dramatic increase in the relative mRNA expressions of *GFP* and *R44* genes in the RCMV-infected brains, from E16 / E17 to P1.

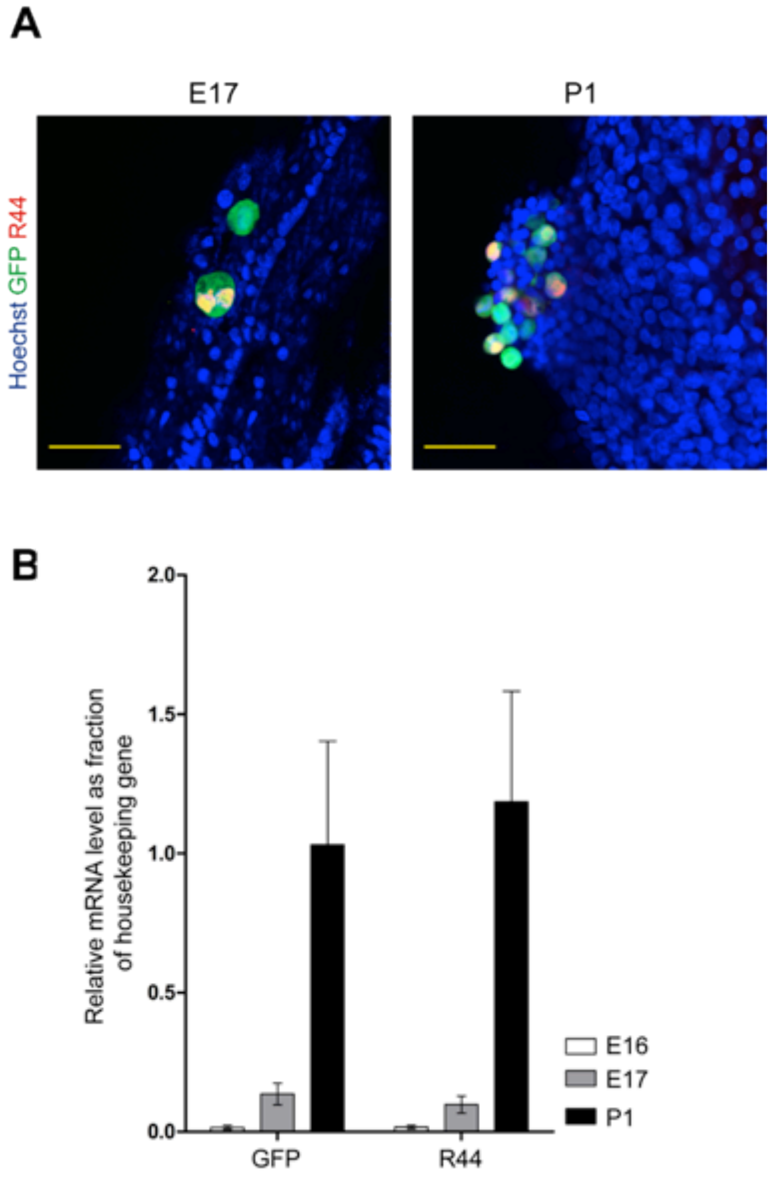

Figure C. Colocalization of RCMV-infected brain cells with CD45 and Iba1 markers at P1.

The subventricular zone of the lateral ventricles (A) and the choroid plexi (B) of the RCMV-infected brains were analyzed at P1 after immunohistochemistry on coronal slices, using confocal microscopy after GFP-expressing RCMV infection at E15. Most GFP+ (infected) cells (green) showed expression of CD45 (red) and Iba1 (white). For each brain area, the bottom pictures represent high magnifications (scale bar: 50 μm) of the corresponding boxed area taken from the corresponding top picture (scale bar: 100 μm). Arrows indicate typical examples of colocalizations of GFP, CD45, and Iba1 signals.

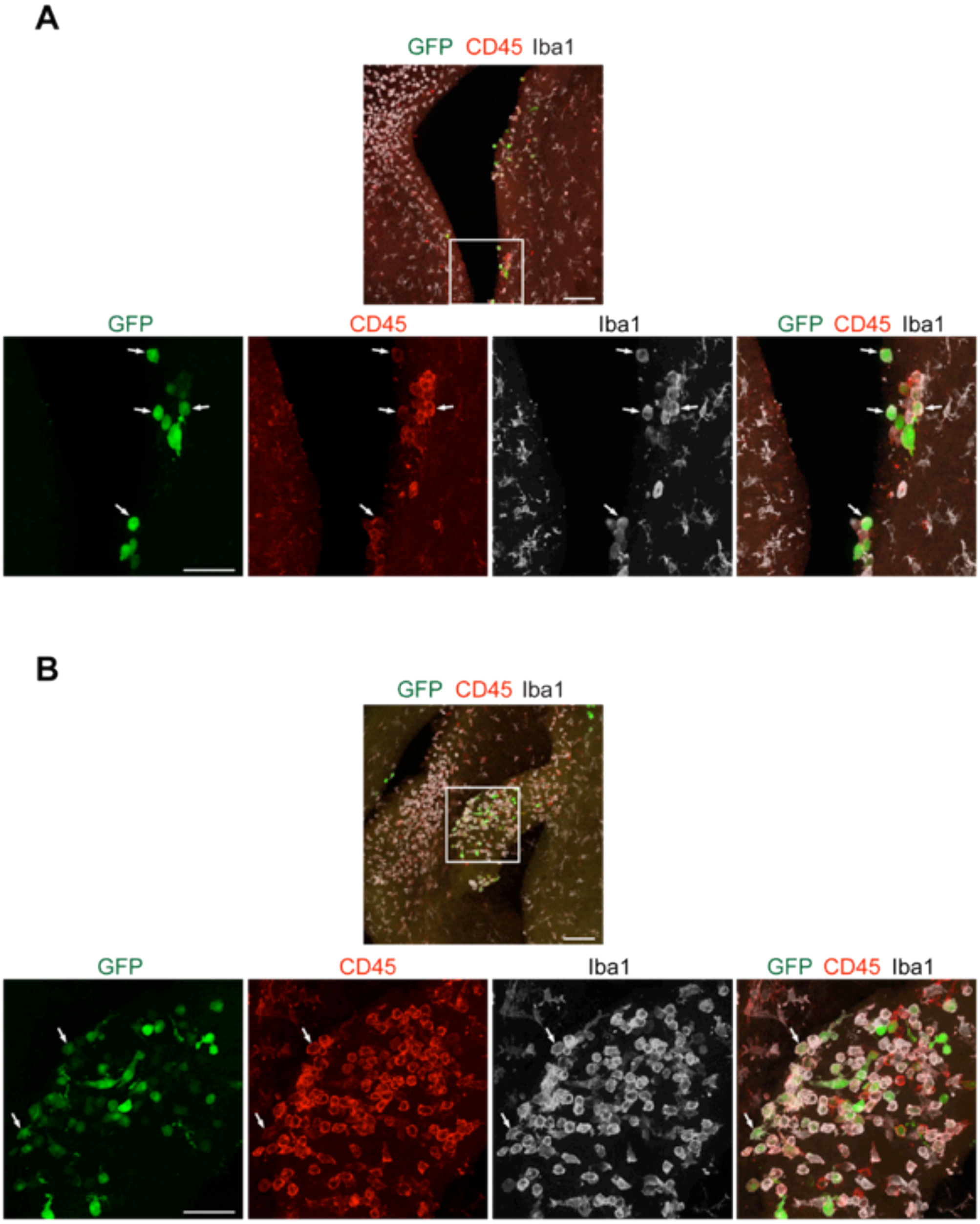

Figure D. Colocalizations of RCMV-infected brain cells with NG2, S100beta and AQP1 markers at P1.

RCMV-infected brains were analyzed at P1 after immunohistochemistry on coronal slices, using confocal microscopy after GFP-expressing RCMV infection at E15. Some RCMV-infected cells (GFP, green) showed (A-C) coexpression of CD45 (red) and NG2 (white) in the subventricular zone of the lateral ventricles, or (D) of S100-beta (red) or (E) of AQP1 (red) in the choroid plexus. (B) and (C) represent high-magnification pictures of the corresponding boxed areas shown in (A). Arrows represent typical colocalizations. Arrowheads (B) point to GFP+ cells that colocalize with CD45 but not with NG2. Scale bars (A): 100 μm ; (B-E): 50 μm.

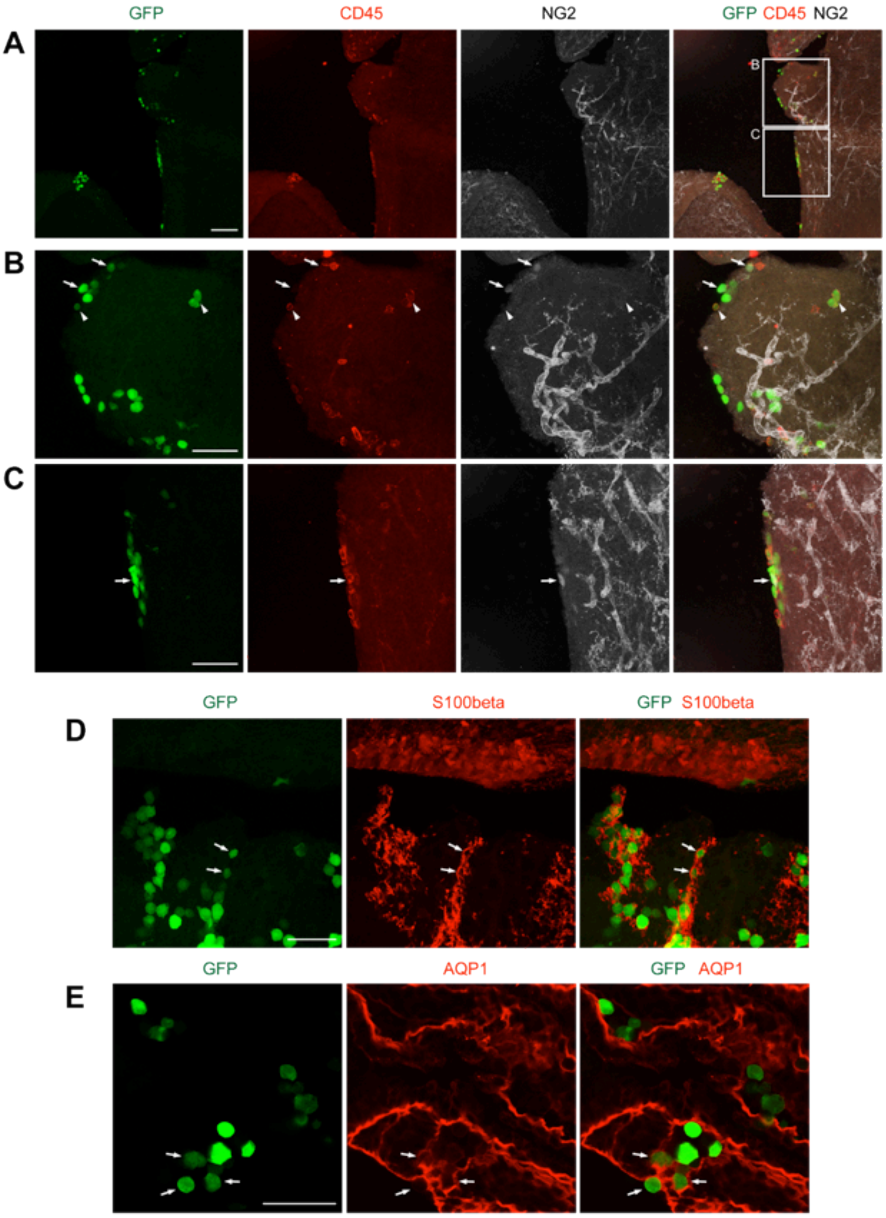

Figure E. Lack of infection and altered morphology of radial glial cells in RCMV-infected brains.

RCMV-infected brains were analyzed at P1 after immunohistochemistry on coronal slices, using confocal microscopy after GFP-expressing RCMV infection at E15. (A) Orthogonal view of RCMV-infected cells that were detected closely adjacent to radial glial cells (nestin+; red) in the ventricular zone of lateral ventricles. No colocalization of GFP and nestin was observed. (B) The morphology of the radial glia was analyzed at P1 in mock (MEM, top) and RCMV-infected (bottom) brains. Note that the altered structure of radial glia (Vimentin, red) was observed (arrows) only in the presence of closely adjacent RCMV-infected cells (GFP, green) whereas the remaining ventricular walls remain unaltered. For each condition small pictures on the right represent magnifications of the corresponding boxes (dorsal telencephalon and ventral telecenphalon, respectively) taken from the left pictures of the lateral ventricles. Hoechst (blue): nuclear staining. White scale bar: 200μm; yellow scale bar: 30 μm.

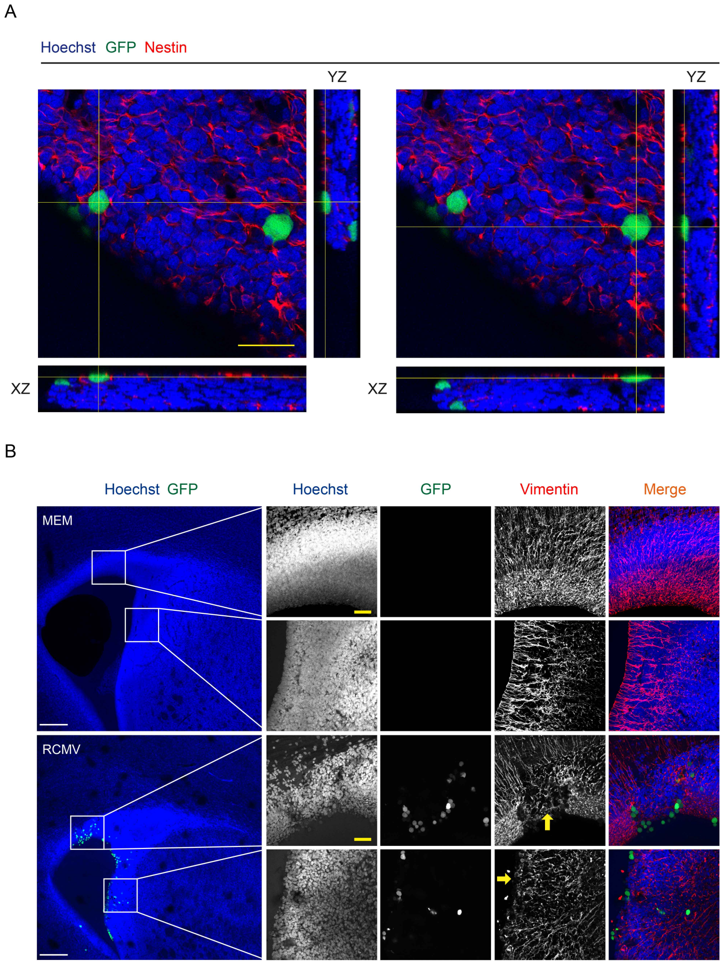

Figure F. Dysexpression of various cytokines and chemokines genes upon RCMV infection of the developing brain.

Bar graphs represent log2-fold changes values in RCMV-infected brains (n=8 for each stage: E16, E17 and P1) *vs* control (MEM) brains (n=8 for each stage). Relative quantification was performed using *RPL-19* as reference gene. Data were analyzed at each stage with Mann-Withney test with Bonferroni correction for multiple testing to check for statistically significant differences between RCMV and control conditions (*p < 0.0045, **p < 0.0009)..

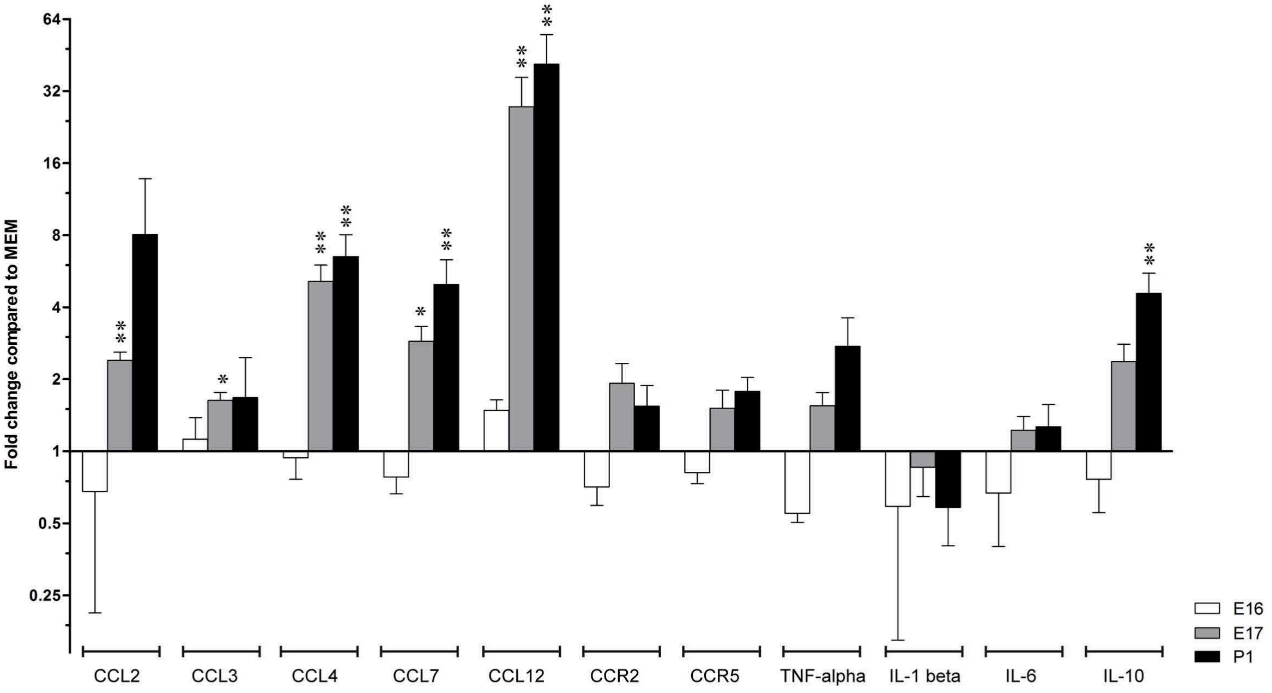

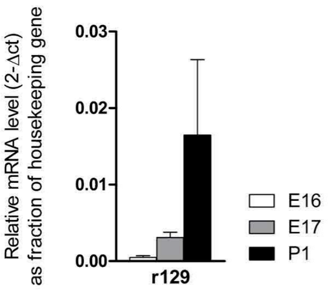

Figure G. Evolution of cell distribution from E17 to P1 in CMV-infected brains by flow cytometry analysis.

Total leukocytes (CD45 events) were gated for CD45 and either Cd11b/c or CD11b expressions and further characterized for CD3 and RT1B or for CCR2 expressions, respectively, as reported in Fig. 4 (E17) and Fig. 5 (P1). For each cell type, relative quantification was done by normalizing the proportion of cells to the average value of the control situation at the corresponding developmental stage. Data were analyzed with Mann-Withney test to check for statistically significant differences between E17 and P1 (ns: non significant; **** $p < 0.0001$).

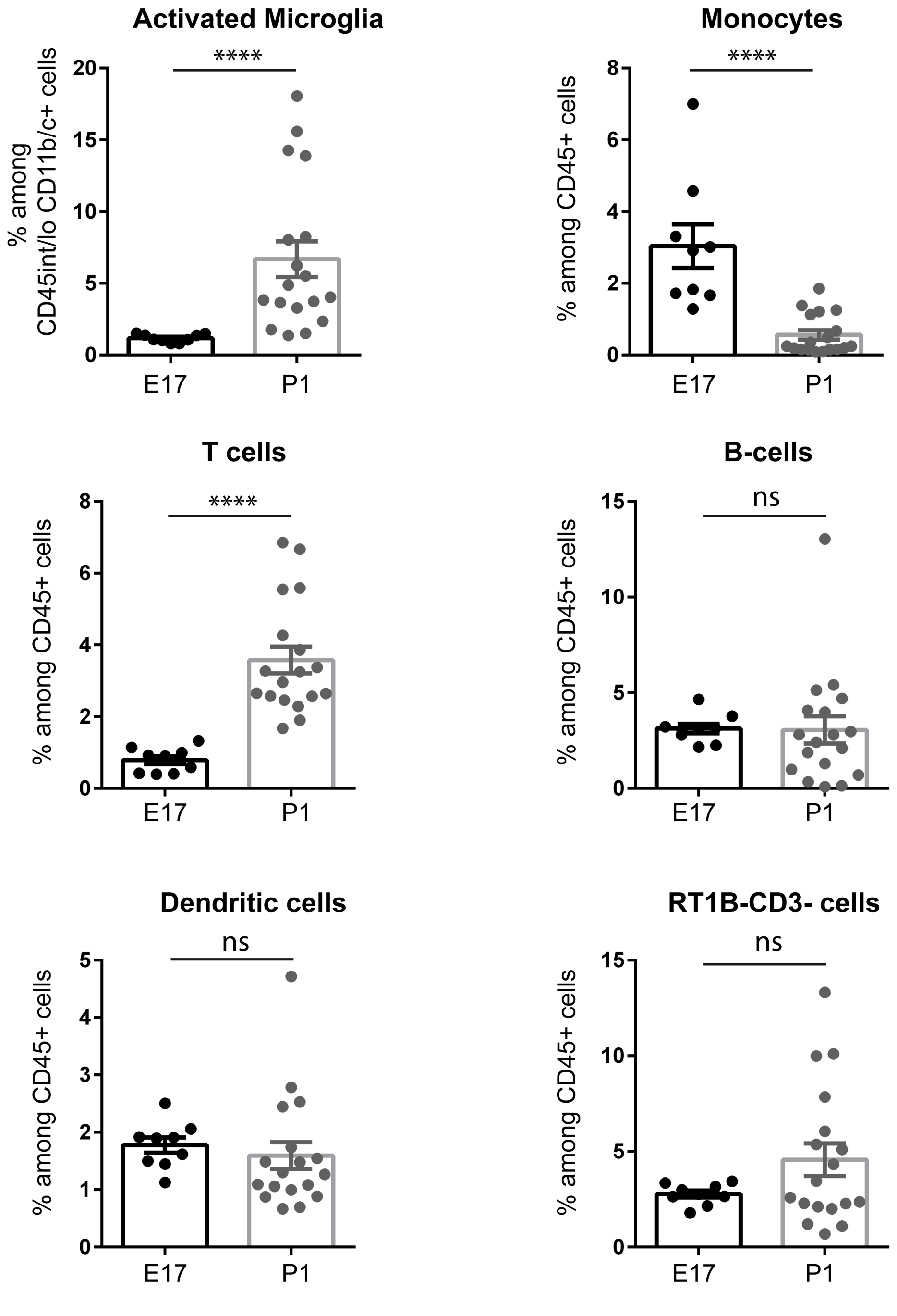

Table A. List of antibodies used in immunohistochemistry and flow cytometry.

Antibody	Dilution	Supplier
Mouse monoclonal anti-RCMV Rmab-8	1:250	G. Grauls (Department of Medical Microbiology, Maastricht University, The Netherlands)
Rabbit polyclonal anti Iba-1	1:500	Wako chemicals USA
Mouse monoclonal anti rat CD68 clone ED1	1:200	Merck Millipore
Mouse monoclonal anti-Vimentin	1:300	Merck Millipore
Mouse monoclonal anti-NeuN	1:500	Merck Millipore
Rabbit polyclonal anti Ki-67	1:300	Merck Millipore
rabbit polyclonal anti aquaporine-1	1:500	Merck Millipore
Mouse monoclonal anti CD45	1:100	BD Biosciences
Mouse monoclonal anti-Nestin	1:300	BD Biosciences
Mouse anti rat CD45-V450	1:25	BD Biosciences
Mouse anti rat CD11b-APC	1:100	BD Biosciences
Mouse anti rat CD11b/c-PE-Cy7	1:100	BD Biosciences
Mouse anti rat CD32 (FcγII receptor)	-	BD Biosciences
Mouse anti rat CD3-BV605	1:200	BD Biosciences
Mouse anti rat RT1B-PE	1:400	Biolegend
Mouse anti CCR2-PE	1:100	R&D Systems
Rabbit polyclonal anti S100	1:500	Dako
Rabbit polyclonal anti NG2	1:200	Merck Millipore

Table B. List of primer sequences.

Name	Sequence	
RPL19	Forward	CCAAGGAAGCACGAAAGC
	Reverse	CCTCCTTGGACAGAGTCTTGA
CCL2 (MCP-1)	Forward	CGGCTGGAGAACTACAAGAGA
	Reverse	TCTCTTGAGCTTGGTGACAAATA
CCL3 (MIP1α)	Forward	TCCACGAAAATTCATTGCTG
	Reverse	AGATCTGCCGGTTTCTCTTG
CCL4 (MIP1β)	Forward	CTCTGCGTGTCTGCCTTCT
	Reverse	GTGGGAGGGTCAGAGCCTAT
CCL7	Forward	TGAAGCCAGCCTTCTCTCTC
	Reverse	TGACATAGCAGCAAGTGGATG
CCL12	Forward	CCTTCTTTGCCTTCTGCTCA
	Reverse	GGATCTTCTGCTTAGCGACATT
CCR2	Forward	AAGAAGTATCCAAGAGCTTGATGAG
	Reverse	TCACCATCATCATAGTCATACGG
CCR5	Forward	GAAGGTGAGACATCCGTTCC
	Reverse	TCGACCCTTGAAAATCCATC
TNF-α	Forward	CAAACCACCAAGCAGAGGAG
	Reverse	GTGAGGAGCACATAGTCGGG
IL-6	Forward	AAAGCCAGAGTCATTCAGAGCA
	Reverse	AGGTTTGCCGAGTAGACCTCAT
IL-1β	Forward	TGTGATGAAAGACGGCACAC
	Reverse	CTTCTTCTTTGGGTATTGTTTGG
IL-10	Forward	AGTGGAGCAGGTGAAGAATGA
	Reverse	TCATGGCCTTGTAGACACCTT
r129	Forward	CGGACCTCAGAACGGACATAC
	Reverse	TCTCTGCAGGATAGTTGGATCTTG

Table C. Absolute numbers of GFP+ cells as determined by cell counting in various brain areas of interest at E17, E20 and P1 (see also Fig. 1).

Each line corresponds to a single brain. For the sake of clarity, brains were given arbitrary numbers. OB: olfactory bulbs; LV: lateral ventricles; CP: choroid plexi; Th: thalamic area; Hyp: hypothalamic area. ND: not determined.

		OB	LV	CP	Th	Hyp
E17	B1	0	4	5	8	2
	B2	0	6	3	2	7
	B3	1	5	14	1	0
	B4	2	3	15	0	0
	B5	0	4	39	3	1
	B6	1	5	12	0	0
	B7	2	4	14	1	4
	B8	0	ND	ND	9	0
	B9	1	ND	ND	1	1
	B10	0	ND	ND	0	1
	B11	0	ND	ND	2	3
	Mean	0,64	4,45	14,57	2,45	1,73
	SEM	0,24	0,40	4,44	0,95	0,66
E20	B1	4	6	2	18	27
	B2	2	21	32	59	39
	B3	0	5	1	137	86
	B4	9	23	6	88	126
	B5	0	30	20	51	17
	B6	7	15	8	22	91
	B7	4	ND	ND	97	21
	B8	5	ND	ND	71	234
	B9	8	ND	ND	112	43
	B10	3	ND	ND	ND	ND
	B11	15	ND	ND	ND	ND
	Mean	5,18	16,67	11,50	72,78	76,00
	SEM	1,33	4,04	4,95	13,30	23,32
P1	B1	37	33	49	119	94
	B2	1	210	97	491	141
	B3	8	86	99	556	467
	B4	2	53	36	285	175
	B5	1	97	112	59	215
	B6	2	226	110	302	197
	B7	26	33	264	ND	ND
	B8	9	256	96	ND	ND
	B9	2	279	224	ND	ND
	B10	14	283	157	ND	ND
	B11	ND	363	ND	ND	ND
	B12	ND	539	ND	ND	ND
	B13	ND	530	ND	ND	ND
	Mean	10,20	229,79	124,43	301,87	214,95
	SEM	3,89	47,85	22,73	80,27	53,45

Table D. Absolute numbers of GFP+ cells and of GFP+ Iba1+ cells as determined by cell counting in the choroid plexi and in the lateral ventricles of RCMV-infected brains at E17, E20 and P1 (see also Fig. 2A).

Each column corresponds to a single brain. For the sake of clarity, brains were given arbitrary numbers. ND: not determined.

		Choroid Plexus							Lateral ventricle						
		B1	B2	B3	B4	B5	B6	B7	B1	B2	B3	B4	B5	B6	B7
E17	Number of GFP+ cells	5	3	14	15	39	12	14	4	6	5	3	4	5	4
	Number of GFP+ Iba1+ cells	1	3	7	8	11	11	7	4	5	3	3	3	2	3
	% of GFP+Iba1+ cells	20	100	50	53	28	92	50	93	82	57	96	81	40	75
E20	Number of GFP+ cells	2	32	1	6	20	8	ND	6	21	5	23	30	15	13
	Number of GFP+ Iba1+ cells	2	15	1	5	17	3	ND	5	4	1	8	12	13	11
	% of GFP+Iba1+ cells	100	47	100	83	85	38	ND	83	19	20	35	40	87	85
P1	Number of GFP+ cells	264	96	224	157	ND	ND	ND	256	279	283	363	539	530	ND
	Number of GFP+ Iba1+ cells	214	67	117	96	ND	ND	ND	154	237	269	246	502	493	ND
	% of GFP+Iba1+ cells	81	70	52	61	ND	ND	ND	60	85	95	68	93	93	ND

Table E. Absolute numbers of Iba1+ cells and of Iba1+ Ed1+ cells as determined by cell counting at E17 (see also Fig. 3A) and at P1 (see also Fig. 3B).

Each column corresponds to a single brain. For the sake of clarity, brains were given arbitrary numbers. ND: not determined.

			B1	B2	B3	B4	B5	B6	B7	B8	B9	Mean	SEM
E17	MEM	Number of Iba1+ cells	24,0	23,0	30,0	58,0	40,0	60,0	20,0	ND	ND	36,4	6,3
		Number of Iba1+ED1+ cells	2,0	2,0	12,0	11,0	9,0	8,0	5,0	ND	ND	7,0	1,5
		% of Iba1+ED1+ cells	8,3	8,7	40,0	19,0	22,5	13,3	25,0	ND	ND	19,5	4,2
	CMV	Number of Iba1+ cells	135,0	157,0	360,0	48,0	57,0	152,0	205,0	ND	ND	159,1	39,6
		Number of Iba1+ED1+ cells	28,0	104,0	176,0	21,0	24,0	55,0	81,0	ND	ND	69,9	21,3
		% of Iba1+ED1+ cells	20,7	66,2	48,9	43,8	42,1	36,2	39,5	ND	ND	42,5	5,2
P1	MEM	Number of Iba1+ cells	174,0	354,0	524,0	209,0	228,0	263,0	185,0	173,0	ND	263,8	42,8
		Number of Iba1+ED1+ cells	16,0	22,0	31,0	17,0	20,0	16,0	13,0	11,0	ND	18,3	2,2
		% of Iba1+ED1+ cells	9,2	6,2	5,9	8,1	8,8	6,1	7,0	6,4	ND	7,2	0,5
		% of Amoeboid cells	21,1	8,5	11,5	23,9	26,3	7,6	21,9	13,7	26,9	16,8	2,6
	CMV	Number of Iba1+ cells	572,0	640,0	755,0	682,0	1279,0	1128,0	843,0	732,0	ND	828,9	87,7
		Number of Iba1+ED1+ cells	126,0	213,0	186,0	147,0	170,0	330,0	156,0	173,0	ND	187,6	22,3
		% of Iba1+ED1+ cells	22,0	33,3	24,6	21,6	13,3	29,3	18,5	23,6	ND	23,3	2,2
		% of Amoeboid cells	52,4	44,5	77,5	61,6	57,5	53,2	89,0	51,2	51,4	60,9	5,3
